# Therapeutic Efficacy of Polymeric Biomaterials in Treating Diabetic Wounds—An Upcoming Wound Healing Technology

**DOI:** 10.3390/polym15051205

**Published:** 2023-02-27

**Authors:** Weslen Vedakumari Sathyaraj, Lokesh Prabakaran, Jayavardhini Bhoopathy, Sankari Dharmalingam, Ramadoss Karthikeyan, Raji Atchudan

**Affiliations:** 1 Faculty of Allied Health Sciences, Chettinad Hospital and Research Institute, Chettinad Academy of Research and Education, Kelambakkam 603103, Tamil Nadu, India; 2 Department of Biotechnology, College of Science and Humanities, SRM Institute of Science and Technology, Kattankulathur 603203, Tamil Nadu, India; 3 School of Pharmacy, Sri Balaji Vidyapeeth, SBV Campus, Pillaiyarkuppam, Puducherry 607402, Tamil Nadu, India; 4 School of Chemical Engineering, Yeungnam University, Gyeongsan 38541, Republic of Korea; 5 Department of Chemistry, Saveetha School of Engineering, Saveetha Institute of Medical and Technical Sciences, Chennai 602105, Tamil Nadu, India

**Keywords:** diabetes, polymers, biomaterials, scaffolds, wound dressings

## Abstract

Diabetic wounds are one of the serious, non-healing, chronic health issues faced by individuals suffering from diabetic mellitus. The distinct phases of wound healing are either prolonged or obstructed, resulting in the improper healing of diabetic wounds. These injuries require persistent wound care and appropriate treatment to prevent deleterious effects such as lower limb amputation. Although there are several treatment strategies, diabetic wounds continue to be a major threat for healthcare professionals and patients. The different types of diabetic wound dressings that are currently used differ in their properties of absorbing wound exudates and may also cause maceration to surrounding tissues. Current research is focused on developing novel wound dressings incorporated with biological agents that aid in a faster rate of wound closure. An ideal wound dressing material must absorb wound exudates, aid in the appropriate exchange of gas, and protect from microbial infections. It must support the synthesis of biochemical mediators such as cytokines, and growth factors that are crucial for faster healing of wounds. This review highlights the recent advances in polymeric biomaterial-based wound dressings, novel therapeutic regimes, and their efficacy in treating diabetic wounds. The role of polymeric wound dressings loaded with bioactive compounds, and their in vitro and in vivo performance in diabetic wound treatment are also reviewed.

## 1. Introduction

Diabetes is a metabolic disorder that ranks as one of the top ten reasons for death among the global population. The International Diabetes Federation (IDF) has reported 463 million diabetic cases in 2019, and this count is suspected to grow to 578 million in 2030 [1,2]. Diabetes mellitus (DM) occurs when the pancreas fails to secrete the necessary amount of insulin required to maintain a normal blood sugar level in the human body. A drastic rise in blood sugar level impairs the process of wound healing, and results in chronic non-healing wounds, which may lead to hospitalization or lower extremity amputation [3]. In diabetic patients, the different phases of wound healing are hindered by various factors such as stalled expression of growth factors, metabolic insufficiency, and reduced physiological response, which prolong the time required for wound recovery [1,3] Diabetes is also connected with different types of illnesses such as chronic kidney failure, cardiovascular disease, stroke, and peripheral neuropathy [4]. Moreover, changes in motor and sympathetic functions may result in physical deformation of the feet due to extreme skin dehydration and wound formation [1]. A recent report states that almost 50 to 70% of all limb amputations are due to diabetes, and one leg is removed every thirty seconds among patients suffering from diabetes [3,5]. The management of diabetic wounds using polymer-based dressing materials has gained a lot of attention among clinicians due to their beneficial properties such as significant antibacterial, mechanical, and wound healing properties [3]. This review highlights the recent advancements in natural and synthetic polymer-based biomaterials for treating diabetic wounds.

## 2. Wound Healing—Physiology

**Figure 1:**
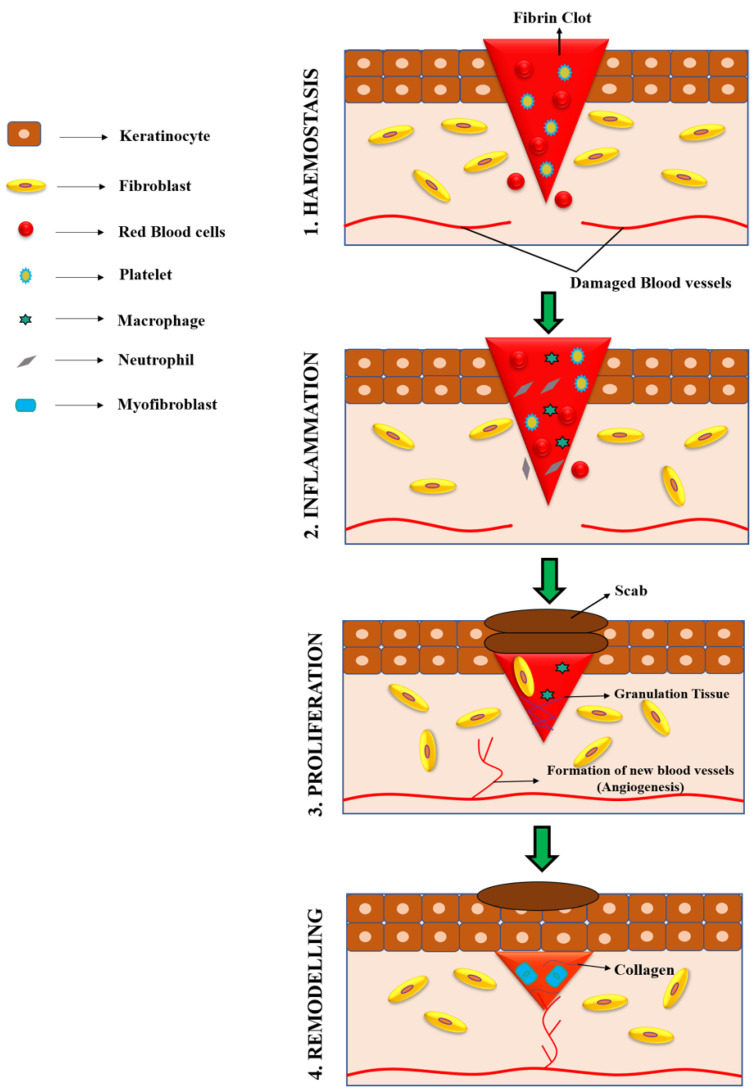
Four different phases of wound healing.

Wound healing is an intricate biological process that occurs when there is a loss of integrity in skin or body tissues [6]. Wound healing requires the involvement of different types of cells, growth factors, enzymes, and various components of the extracellular matrix for repairing and restoring damaged tissues and organs [3]. It occurs in four distinct stages: haemostasis, inflammation, proliferation, and remodelling (Figure 1).

These four phases must proceed in an ordered fashion to avoid interruptions or delays in wound closure [7]. Haemostasis is a process that begins immediately after an injury to stop bleeding, and results in the formation of blood clots. During this event, platelets aggregate at the wounded site owing to the interaction with proteins such as collagen and fibronectin. Soluble fibrinogen is converted into insoluble fibrin to arrest bleeding. The area surrounding the clot and damaged tissue produces growth factors and pro-inflammatory cytokines that aid in efficient wound healing. When the bleeding has stopped, the inflammatory phase is initiated, and this involves the migration of leucocytes to the injured site to eliminate debris and infectious microorganisms [8]. This phase is characterized by the sequential infiltration of distinctive kinds of cells such as macrophages, neutrophils, and lymphocytes, which protect the wounded site from infections [9,10,11]. Macrophages play a vital role in all the stages of wound healing [12,13] as they release cytokines that are responsible for inflammation, activation of leukocytes, and clearance of apoptotic cells. As soon as apoptotic cells are cleared, macrophages are transformed into a pro-regenerative state that activates fibroblasts and keratinocytes, resulting in the regeneration of tissues. The proliferative phase is overlapped with the inflammatory phase, which leads to the proliferation and migration of epithelial cells. Fibroblasts and epithelial cells perform a vital task in the formation of collagen and granulation tissue at the site of the wound. The main components of the extracellular matrix—collagen, glycosaminoglycans, and proteoglycans—are synthesized by fibroblasts, and they play a vital role in the healing of the wound. At the end of the proliferative phase, the wound healing process moves into the final remodelling phase, which is characterized by the formation of granulation tissue [9,14].

## 3. Wound Healing in Diabetes

**Figure 2:**
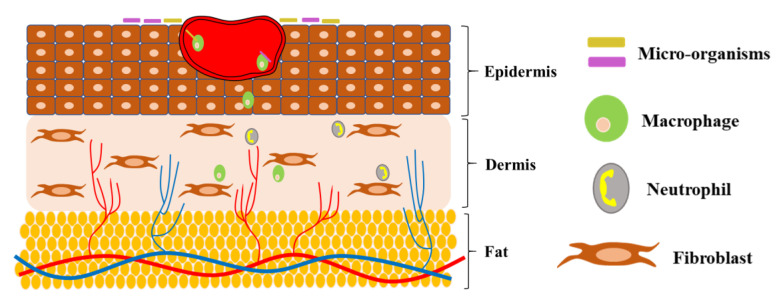
Diagrammatic representation of normal wound healing.

**Figure 3:**
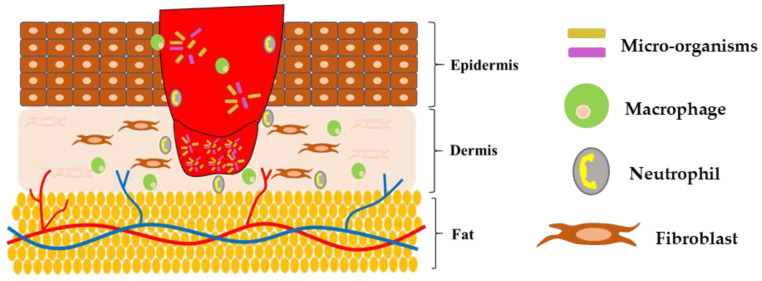
Impaired or delayed wound healing under diabetic conditions.

The normal phases of wound healing are disrupted due to diabetes (Figure 2). Diabetic wounds (Figure 3) continue to persist in the inflammatory phase, and the development of matured granulation tissues is inhibited by the hindering of the initiation of the proliferative phase in wound healing [3,15]. Intrinsic and extrinsic factors are involved in impairing the healing of diabetic wounds. Continuous mechanical stress and recurrent trauma can further deteriorate the healing process and result in ulcer formation [16,17]. Diabetic wound healing is delayed due to various causes such as neuropathy, poor immunity, microbial infection, oxygen deficit, and minimal activity of growth factors [3,7,18]. Numerous cells such as macrophages, neutrophils, fibroblasts, lymphocytes, keratinocytes, mast cells, and endothelial cells are actively involved in the normal wound healing process. Several growth factors and cytokines are secreted by these cells, which perform a key role in accelerating wound healing. Increased blood sugar level alters macrophage polarization, which serves as one of the chief causes for impaired diabetic wound healing. Events such as continuous secretion of pro-inflammatory cytokines, reduced angiogenic response [7], decreased activity of neutrophils, macrophages, and fibroblasts, were observed in diabetic wounds [19,20]. Diabetic wounds may also result in sensory disability towards temperature, pressure, and lesions. Lack of pain and abnormal vasodilator autoregulation together aggravate the process of wound healing [3]. Diabetic wounds may limit physical movement and cause psychiatric stress and depression [15].

## 4. Types of Diabetic Wound Dressings

Wound dressings quicken the process of wound healing by allowing water transmission, providing a moist atmosphere, and aiding in improved granulation and re-epithelialization. They can be incorporated with therapeutic molecules or anti-microbial agents for efficient treatment of wounds [3]. The most commonly used diabetic wound care products available on the market are Comfeel, Granuflex, and Duoderm. However, serious concerns are raised about their use in treating infected wounds, as they may cause maceration to the surrounding tissues that are present around the wound. Intrasite Gel and Aquaform are two types of hydrogels that are used in wound treatment, but their use in treating diabetic foot lesions are restricted in individuals with limb ischemia [21]. Even though there are different types of commercially available diabetic wound dressings, the percentage of exudate absorption varies between them, which demands the development of new materials for treating different types of diabetic wounds. The new materials developed must hold a perfect balance between therapeutic molecules and antibiotics that are used to reduce healing time and the chance of formation of new ulcers [22].

**Figure 4:**
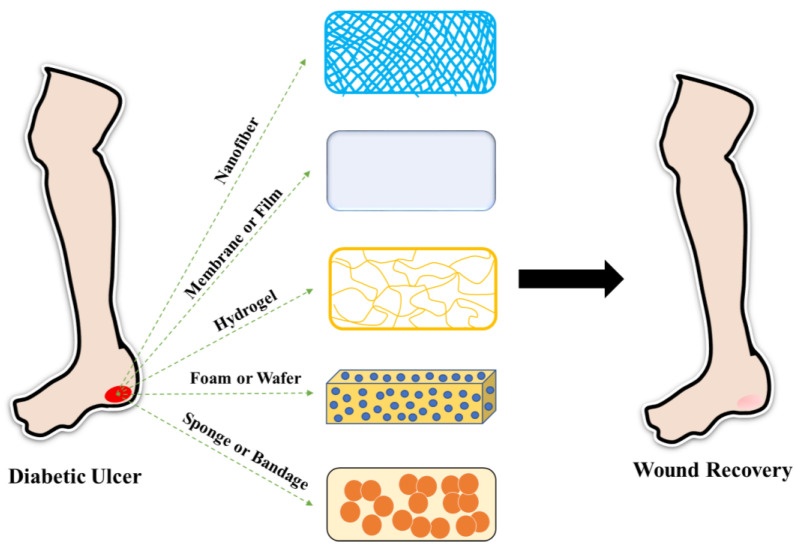
Different types of wound dressings used for diabetic wound treatment.

The different forms of wound dressings (Figure 4) are as follows.

(i) Films are transparent, sticky materials that are widely used in the field of wound treatment. Their transparent nature assists in monitoring the healing of wounds without disturbing the injured site and dressing material [23]. They allow the permeation of gases including oxygen, water vapor, and carbon dioxide between the wounded site and the environment [24]. Film-based dressing materials have various benefits such as high flexibility and elasticity, and ease in fabricating them in the desired size and shape based on the application [25].

(ii) Hydrogels are commonly used in tissue engineering and wound recovery. They are formulated by the physical or chemical cross-linking of natural or synthetic polymers. Due to their three-dimensional polymeric network, they have the ability to absorb a high quantity of water molecules when compared with their dry weight. This property makes them superior among all wound dressing materials as they can retain excessive moisture content at the wounded site [26]. They can be fabricated in various forms and sizes, and loaded with anti-microbial substances, cells, and growth factors for reducing the time required for wound closure [27]. The ability of hydrogels to maintain a moist milieu helps in promoting granulation and re-epithelization, which, in turn, results in the regeneration of tissues.

(iii) Nanofibrous dressings are a group of nanofibers with sizes ranging from nanometres (nm) to micrometers (μm) [28]. Various strategies are employed to fabricate nanofibers, but the electrospinning technique is one of the most extensively utilized methods due to its enormous merits such as cost-effectiveness, ease, versatility, control of porosity, and tuning of mechanical properties of nanofibers. Once applied on the wound, the nanofibers can be removed easily without causing any damage to the applied site [29,30,31]. They can be loaded with various bioactive molecules for treating non-healing wounds [32]. Nanofibers have the ability to imitate the native extracellular matrix. They also offer an appropriate environment for cell proliferation and adhesion for rapid healing of wounds [33].

(iv) Foam is a type of wound dressing material that is composed of both hydrophilic and hydrophobic foam with bioadhesive boundaries [34]. The hydrophobic portion prevents unnecessary entry of liquids into the wound bed but permits gaseous exchange and water vapor permeation. The advantages of using a foam-based wound dressing are that they can maintain appropriate moisture content and absorb excess volume of wound exudates [35]. Based on the thickness of the wound, foam has the ability to absorb different amounts of wound exudates [36]. However, foams are inappropriate for dry wounds with fewer exudates [37].

(v) Wafer-based wound dressings are extremely porous freeze-dried polymers that have similar characteristics to those of foams. Wafers absorb the exudate of a wound and transform it into a gel or viscous solution that provides a moist atmosphere [38]. A few polymers including xanthan gum and sodium alginate have been used for the fabrication of wafers for biomedical applications [39]. The wound exudate-absorbing property of wafers helps in reducing fluid collection and microbial infection, which, in turn, aids in the quick recovery of wounds.

(vi) Sponges are soft and flexible with interconnected pores. Due to their porous nature, they have excellent swelling ability, which is an ideal feature of a wound dressing material [40]. Different kinds of sponges have been fabricated with different types of polymers for delivering therapeutic molecules for the efficient treatment of diabetic wounds. Sponges have been proven to help in cell migration and prevent microbial infection at the wound site [3]. Due to the presence of interconnected pores, sponges enhance the migration of fibroblasts, which results in faster closure of wounds.

## 5. Polymeric Biomaterials

Although there have been numerous advancements in the area of wound healing, the treatment of chronic wounds continues to be a major problem in patients suffering from diabetic foot ulcers and other major injuries [41]. An ideal wound dressing material must possess special features such as absorbing wound exudates, aiding in appropriate exchange of gas, and protecting from microbial infections. They must also support in the synthesis of biochemical mediators such as cytokines and growth factors that are essential for the proper healing of wounds [42]. Bioactive compounds extracted from natural sources have been investigated to understand their role in accelerating the process of healing diabetic wounds. The study of natural materials in the form of wound dressing has gained special attention because of their potency in inducing the formation of new tissues. Synthetic polymers are also extensively used in treating diabetic wounds as they exhibit excellent mechanical, bioinert, and biocompatible characteristics. Both natural and synthetic polymeric biomaterials are considered as satisfactory wound dressings due to their exceptional properties such as increased wound healing efficacy, less/no immunogenicity, good mechanical strength, and biocompatibility. Table 1 shows the different types of wound dressing materials prepared using both natural and synthetic polymers. Herein, we discuss the various wound dressing polymeric biomaterials that are used for treating diabetic wounds.

### 5.1. Natural Polymers

Natural biomaterials are considered to be suitable candidates for preparing wound dressing material due to their exceptional properties such as less/no immunogenicity and good biocompatibility. They also serve as satisfactory matrices for cells that play imperative roles in the process of wound healing. Some of the widely accepted natural products extracted from natural sources that are widely used as wound dressing material are collagen, gelatin, fibrin and silk proteins.

#### 5.1.1. Collagen

Collagen provides integrity to human skin and serves as a principal component of the extracellular matrix (ECM) [96]. It is abundantly present in bones, ligaments, and tendons. It has distinctive properties such as excellent biocompatibility, thermal stability, mechanical strength, and low immunogenicity [97,98]. Collagen plays a vital role in haemostasis as it interacts with the platelets that are deposited at the site of wound through chemotaxis [99]. It mediates various pro-regenerative physiological interactions that are responsible for wound healing. Collagen is extensively used as a matrix for wound treatment and tissue regeneration. Collagen is isolated from different types of sources such as bovine, equine and, porcine tissues [100,101]. Although there are 29 types of collagens, type 1 collagen is widely available and can be extracted easily from mammalian connective tissues [102,103]. The most commonly used type 1 collagen is isolated from the tendons of rat tails [104,105]. Bovine collagen is extracted from several tissues such as bone, skin, and the Achilles tendon [106,107]. Collagen is formulated in the form of scaffolds with varying concentrations and pore sizes. These scaffolds can absorb wound exudates, attach onto the wound bed, and provide moist environment [108].

**Figure 5:**
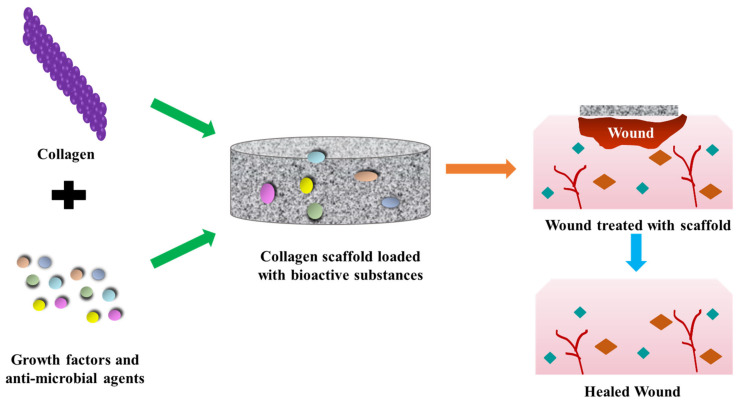
Collagen scaffold loaded with therapeutic molecules for diabetic wound treatment.

Collagen-based scaffolds (Figure 5) are commonly used as a wound dressing material for treating skin burns, foot ulcers, and pressure sores [109]. In certain cases, collagen is combined with other sources such as fibronectin or elastin to improve the fluid-binding property of the scaffolds [110,111]. Collagen is also fabricated in the form of implants that can be used as a support for delivering keratinocytes for skin regeneration [112,113,114]. After implantation, collagen scaffolds are infiltrated by connective tissues that are composed of glycosaminoglycan, new collagen, fibroblasts, and macrophages. Based on the cross-linking percentage, collagen scaffolds are degraded into small peptides within a few weeks of implantation, and replaced by native collagen that is synthesized by fibroblasts [115]. Apligraf^®^—the first tissue-engineered wound dressing material that was approved for treating diabetic ulcers—was made up of two-layered collagen hydrogels loaded with human keratinocytes and fibroblasts [116]. Subsequently, several modifications were carried out by altering the concentration of collagen to improve the mechanical strength of Apligraf^®^. When collagen concentration was increased, there was a significant rise in the proliferation of fibroblasts and stimulation of keratinocyte growth factor, which lead to the faster healing of wounds [117]. At present, collagen-based wound dressing materials are widely accepted in managing full-thickness wounds and skin burns. It is also possible to enhance the activity of collagen by combining it with bioactive therapeutic agents and antimicrobial compounds substances that accelerate the rate of wound closure [118]. Type 1 collagen has the ability to draw growth factors toward the wounded area and quicken the healing and regeneration of damaged tissues [98]. However, in the case of diabetic wounds or diabetic foot ulcers, the epidermis becomes ulcerous, resulting in the deficiency of type 1 collagen. This further delays the proliferation and migration of fibroblasts, which, in turn, prolongs the time required for wound healing [119]. Lee et al. determined the ability of collagen (colladerm) wound dressing in treating diabetic foot ulcers. When patients were treated with collagen dressing every 2–3 days for up to 3 weeks, a significant decrease in the wound area with 73.7% healing of the diabetic wound ulcer was observed. The results demonstrated the safety and efficacy of collagen dressing in faster healing of diabetic foot ulcers [120]. Hauck et al. demonstrated the possible use of hyaluronan/collagen hydrogels loaded with high-sulphated hyaluronan in treating dermal wounds in diabetic mice models. The hydrogel enhanced the healing rate of damaged tissues with decreased inflammation, improved vascularization, and increased pro-regenerative macrophage activation, and hastened the formation of new tissues for wound closure [121]. Shagdarova et al. prepared hydrogels using chitosan, collagen, and silver nanoparticles for treating diabetic injuries/wounds. The hydrogels had a fibrous porous structure with a better swelling ratio. When applied onto diabetic wounds, the hydrogels elevated the expression of genes such as vascular endothelial growth factor, Interleukin 1b, tissue inhibitor of metalloproteinases-1, and transforming growth factor beta 1 [122].

#### 5.1.2. Gelatin

Gelatin is a natural polymer that is obtained from the partial hydrolysis of collagen [123]. Due to its salient features such as availability, biodegradability, biocompatibility, cell-interactivity, and non-toxicity, gelatin is commonly used in the field of biomedicine [124]. When used as a scaffold, gelatin has the ability to absorb water molecules, making it an appropriate candidate for wound dressing material. The main drawback associated with gelatin is its poor stability and mechanical strength. Therefore, to increase its mechanical stability, gelatin is cross-linked with agents such as glutaraldehyde, fructose, dextran, genipin, formaldehyde, and carbodiimides [125]. Samadian et al. developed berberine-loaded cellulose acetate/gelatin electrospun mats as wound dressing for treating diabetic foot ulcers. The fibres had an average diameter of 502 ± 150 nm, and demonstrated antibacterial behaviour against *Staphylococcus aureus* and *Pseudomonas aeruginosa*. The electrospun mats exhibited suitable tensile strength and water uptake potency required for a wound dressing material. A haemolytic assay performed using red blood cells showed that the percentage of haemolysis was significantly low for berberine-loaded cellulose acetate/gelatin electrospun mats when compared with the positive control—water [126]. Yu et al. prepared a paeoniflorin-sodium alginate (SA)-gelatin skin scaffold with a mesh-like structure with uniform pore distribution for treating diabetic wounds. Animal models showed improved deposition of collagen with microvascular regeneration when treated with the skin scaffold, thereby proving their possible use in the field of diabetic wound treatment [127]. Sadeghi et al. prepared biodegradable scaffolds using gelatin and sulphated alginate as skin replacements to accelerate the healing of diabetic wounds. The carbodiimide mode of cross-linking followed by lyophilization was carried out to prepare the scaffolds. Cell culture analysis proved the non-toxicity of the scaffolds, with enhanced cell growth when the quantity of sulphated alginate was increased in the scaffold. Diabetic animal models proved the ability of the scaffold to cure wounds by providing the required environment for faster healing of wounds [128].

#### 5.1.3. Fibrin

Fibrin is obtained from fibrinogen, which is converted in response to tissue injury. It acts as a mesh and forms blood clots to prevent bleeding. Fibrin is extensively used for clinical applications, in the form of sealants and haemostatic agents [129]. When used in the form of scaffolds, it has the ability to deliver inflammatory cells and growth factors that are necessary for wound repair and tissue regeneration [130].

**Figure 6:**
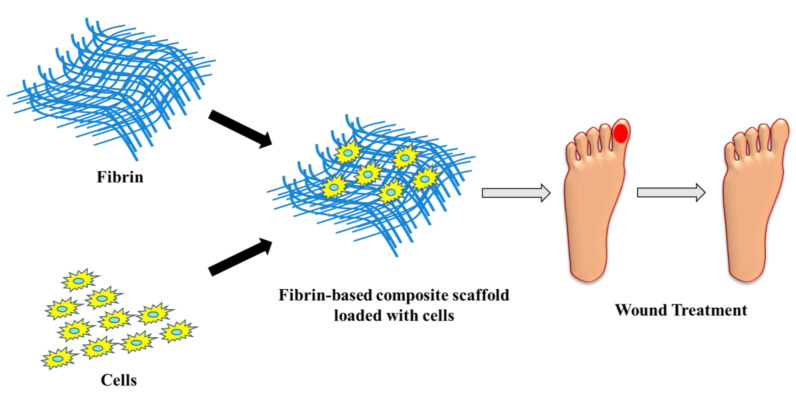
Fibrin-based therapy for wound treatment.

**Figure 7:**
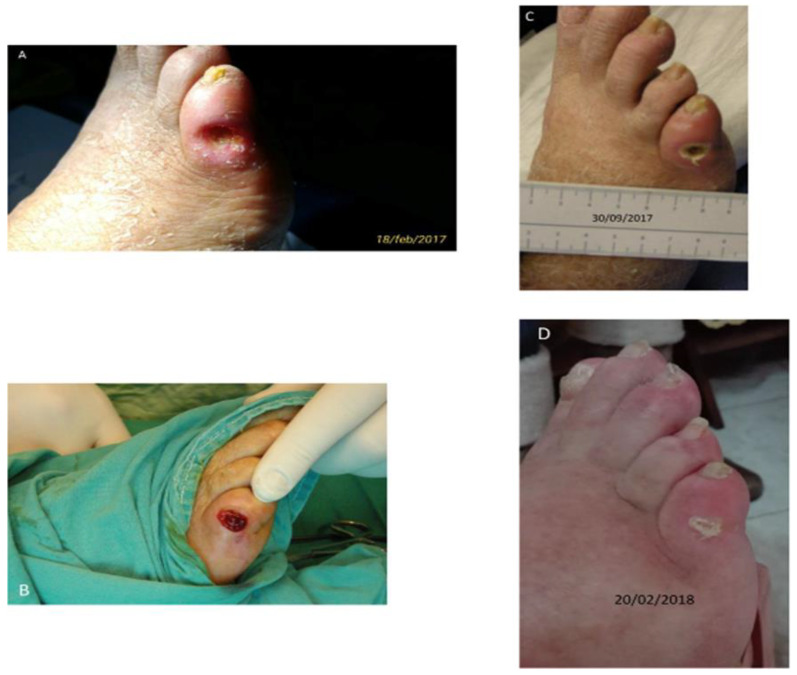
Efficacy of fibrin rich in leukocytes and platelets (FLP) in treating osteomyelitis ulcers in diabetic feet. Reprinted from Ref. [137].

Fibrin serves as a substrate for different types of cells such as platelets, fibroblasts, endothelial cells, and macrophages. It triggers the process of cellular proliferation and new blood vessel formation, thereby leading to efficient healing of wounds. Fibrin can be formulated in different structures such as nanoparticles, hydrogels, scaffolds and films. Fibrin-based cell delivery is widely accepted for treating dermal wounds, as it stimulates neovascularization and the rejuvenation of skin cells [131]. For treating skin burns, fibrin-based hydrogels/films are utilized for the transplantation of keratinocytes to induce fibroblast formation and re-epithelization. Fibrin scaffolds loaded with vascular endothelial factor and fibroblast growth factor enhanced re-epithelization, collagen deposition, and accelerated wound closure in mice with diabetic wounds [132]. Fibrin as a scaffold has the ability to mimic the extracellular matrix and enhance the interaction of cells responsible for tissue regeneration [133,134]. Geer et al. studied the re-epithelializing performance of fibrin using human keratinocytes under in vitro conditions [135]. Falanga et al. demonstrated that when bone marrow-derived mesenchymal stem cells (Figure 6) were entrapped within fibrin, they reduced the time taken for wound closure in acute and chronic wounds [136]. When bone marrow nuclear cells were mixed and injected along with fibrin gel to treat infarcted myocardium, it resulted in neovascularization and increased tissue regeneration. Fibrin-based formulations are highly efficient in curing diabetic wounds. Crisci et al. investigated the efficacy of fibrin rich in leukocytes and platelets (FLP) in treating osteomyelitis ulcers in diabetic feet. FLP was collected from diabetic individuals suffering from osteomyelitis and skin lesions for a minimum period of 180 days. Surgical debridement was carried out to deliver FLP directly into skin lesions of patients, and the development of lesions was assessed periodically. The study report (Figure 7) stated that FLP treatment settled down skin lesions with no indication of microbial infection [137].

Losi et al. employed electrospinning and spray phase-inversion procedures to synthesize bilayered fibrin/poly(ether)urethane scaffolds rich in platelet lysate for treating diabetic wounds. Cell culture experiments performed using L929 mouse fibroblasts proved that the efficacy of two important growth factors—platelet-derived growth factor (PDGF) and vascular endothelial growth factor (VEGF), which play a crucial role in healing chronic wounds—was retained in the electrospun scaffolds. An in vitro release study of PDGF and VEGF from the synthesized scaffolds demonstrated an initial burst release of growth factors within 24 h of study, followed by sustained release for one week. When applied onto full-thickness wounds in diabetic animal models, a significant improvement in wound closure within 2 weeks of treatment was observed. Moreover, improved re-epithelialization and collagen deposition were also witnessed in wounds treated with the scaffolds, thereby proving their potency in healing diabetic wounds/ulcers [138]. Poly(ether)urethane-polydimethylsiloxane/fibrin-based scaffolds containing poly(lactic-co-glycolic acid) nanoparticles loaded with recombinant human vascular endothelial growth factor and basic fibroblast growth factor were fabricated by Losi et al. with the intention of triggering cellular proliferation and accelerating the process of wound healing in genetically diabetic mice. The presence of growth factors in the scaffolds quickened the rate of closure of full-thickness skin wounds on day 15 in diabetic mice. Histological analysis showed extensive re-epithelialization, with increased granulation tissue formation/maturity and collagen deposition, thereby elucidating the efficiency of the prepared scaffolds in treating diabetic wounds [133].

#### 5.1.4. Silk Proteins

**Figure 8:**
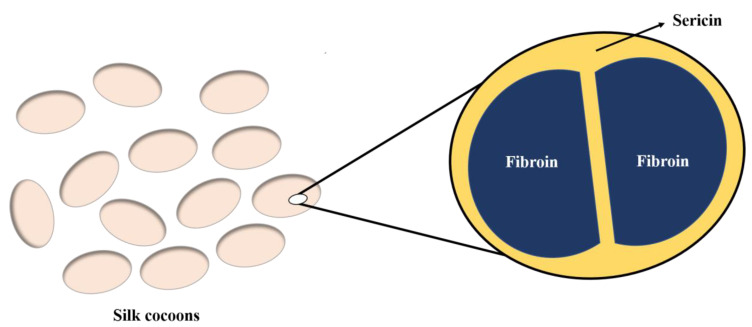
Two different types of silk proteins—silk fibroin and silk sericin.

Silk cocoons are discarded as waste by the silk industry, but they can be used as valuable resources for fabricating wound dressings that can aid in faster healing of wounds. Silk-based biomaterials are extensively used in the field of medicine due to their excellent biocompatibility and biodegradability. The FDA approved product silk voice is a type of scaffold, prepared using reconstituted or solubilized silk protein [139]. Silk protein has the capability to induce cell migration and proliferation, and attract cells such as keratinocytes to the wounded site, thereby accelerating the process of wound healing [140]. Two different types of proteins—silk fibroin and silk sericin—are isolated from the cocoons of silkworms (Figure 8). They are widely used in biomedical applications due to their lower immunogenicity, biodegradability, biocompatibility, moisture absorption, UV resistance, and antibacterial properties [141].

Silk proteins are fabricated in various forms such as films, nanofibers, and sponges for biomedical applications. When used as a wound dressing material, silk proteins have enhanced fibroblast adhesion and lead to faster healing of wounds [142]. The middle and posterior silk glands of silkworm *Bombyx mori* secretes two important proteins, silk fibroin and silk sericin, which are extensively used to accelerate the healing of wounds [143]. Silk fibroin has extraordinary thermal stability and mechanical strength when compared with polymers such as collagen and polylactic acid. In addition, the presence of an RGD peptide sequence promotes the attachment, movement, and proliferation of cells such as keratinocytes, endothelial, epithelial, and glial cells, and osteoblasts for the effective healing of wounds. Porous silk fibroin scaffolds/sponges can be prepared using different types of techniques such as lyophilization, gas forming, and freeze-drying/foaming methods [144]. Liu et al. prepared silk fibroin scaffolds incorporated with neurotensin-loaded gelatin microspheres as a novel therapeutic regime for healing diabetic foot ulcers in diabetic rat models. Macroscopic evaluation of wounds showed significant reduction in wound size on day 14 in the experimental group. In addition, histological and immunofluorescence analyses demonstrated the accumulation of fibroblasts with a substantial expression of collagen at the site of the wound. The prepared scaffolds had a good porosity of approximately 85%, with an average pore size of 40–80 μm [145]. Guan et al. fabricated microneedle patches with multiple features such as anti-microbial, anti-oxidant, and pro-angiogenic properties for targeting diabetic lesions/wounds. The microneedles were constructed using silk fibroin methacryloyl with tremendous biocompatibility and mechanical stability. Two different bioactive molecules—VEGF and Prussian blue nanozymes—were loaded on the tip of microneedles with polymyxin—an anti-bacterial agent—at the base layer of the microneedle patches. The patches played a significant role in treating diabetic skin wounds [146]. Xu et al. prepared electrospun Huangbai liniment-loaded silk fibroin/poly-(L-lactide-co-caprolactone) nanofibers for treating diabetic wounds. The fibres were smooth, without any bead formation when viewed under a scanning electron microscope. The nanofibers exhibited antibacterial activity against *Escherichia coli* and *Staphylococcus aureus.* Cell culture experiments showed enhanced adhesion and proliferation of NIH-3T3 cells when cultured on the nanofibers. Animal experiments using a diabetic mice model proved that the nanofibers had the ability to elevate the synthesis of collagen and expression of the TGF-β signalling pathway, which, in turn, promoted efficient healing of diabetic wounds [147]. Silk sericin is a globular protein with significant features such as non-immunogenicity, good biocompatibility, biodegradability, anti-oxidant properties, and regenerative potency. It serves as a promising biomaterial in the field of medicine. Silk sericin-based hydrogels were prepared using horseradish peroxidase (HRP) and hydrogen peroxide cross-linking methods. A chorioallantoic membrane assay performed using chick embryos showed a minimal rise in the number of new blood vessels in the test group when compared with that of the control. The hydrogel showed collagen deposition and mild induction of superoxide dismutase and catalase in diabetic wounds treated with hydrogel in the mouse model [148]. Samad et al. formulated carboxymethyl cellulose/sericin hydrogels for diabetic wound treatment. The hydrogels had porous morphology, excessive swelling efficacy, and anti-microbial properties. When applied on to full-thickness excision wounds in diabetic rats, the upregulation of collagen deposition and downregulation of pro-inflammatory markers was witnessed, which led to the healing of wounds without any insulin treatment [149].

### 5.2. Synthetic Polymers for Diabetic Wounds

Synthetic polymers are used in combination with natural polymers for treating diabetic wounds because they exhibit excellent mechanical properties. They are used for tissue engineering applications owing to their inert and biocompatible characteristics. Synthetic polymers such as polycaprolactone (PCL), poly(vinyl alcohol) (PVA), Poly(2-hydroxyethyl methacrylate) (pHEMA), polylactide (PLA), and polyglycolic acid (PGA) have been used as scaffolds in tissue engineering and wound healing applications along with natural polymers [3,150,151].

#### 5.2.1. Polycaprolactone (PCL)

**Figure 9:**
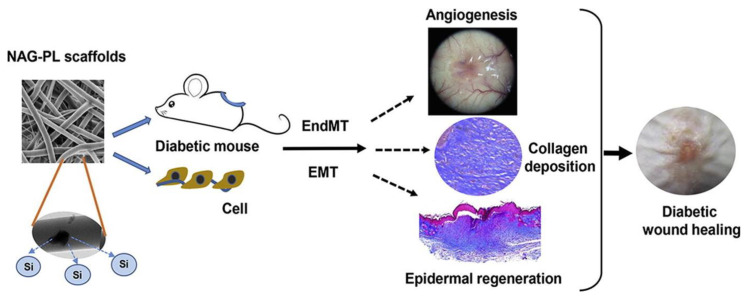
The role of poly (caprolactone)/gelatin nanofibrous scaffolds in treating diabetic wound healing. Si—Silicon ions; NAG-PL—Nagelschmidtite-Poly(caprolactone); EndMT—Endothelial mesenchymal transformation; EMT—Epithelial-to-mesenchymal transition. Reprinted from Acta Biomaterialia, Vol. Number 60; Lv F., Wang J., Xu P., Han Y., Ma H., Xu H., Chen S., Chang J., Ke Q., Liu M., Yi Z.; A conducive bioceramic/polymer composite biomaterial for diabetic wound healing, Pages No. 128–143, 2017 with permission from Elsevier [154].

PCL is a hydrophobic polymer that has a great degradation rate and excellent bioactivity. It is a linear aliphatic semicrystalline polymer. PCL polymer can be modified by changing the molecular weight, crystallinity, or structure using polyethylene glycol and hydrophobic ceramics, or by creating copolymers with PLA and PGA. PCL exhibits reduced cellular attachment owing to its hydrophobicity, which can be altered by modifying its surface with other biomaterials [152]. An organic and inorganic composite scaffold containing two-dimensional nanovermiculite and PCL electrospun fibres for treating diabetic wounds were prepared by Huang et al. The results show that polycaprolactone electrospun fibres with two-dimensional vermiculite nanosheets could significantly improve neo-vascularization, re-epithelialization, and collagen formation in the diabetic wound bed [153]. Amine-terminated block copolymers containing PCL and polyethylene glycol and PCL were electrospun using electrospinning technique by Choi et al. The human epidermal growth factors (EGF) were immobilized on the surface of the nanofibers. Dorsal wounds were created in diabetic animals in order to study the wound healing efficacy of the prepared wound dressing material. Immunohistochemical studies showed that the EGF receptor were highly expressed in the nanofiber-treated groups. The results showed that the prepared nanofibers could be a potential material for treating diabetic wounds [47]. Merrell et al. used PCL nanofibers as drug delivery vehicles. He prepared PCL nanofibers loaded with curcumin and used them as a diabetic wound dressing material. In total, 70% of human foreskin fibroblast cells (HFF-1) cells were viable when treated with the prepared nanofibers. A streptozocin-induced diabetic mouse model were used for the in vivo study, which showed an increased rate of wound closure in animals treated with the nanofibers. The study proved that the prepared nanofibers are bioactive, with anti-inflammatory and antioxidant properties [53]. Lv et al. prepared a PCL/gelatin nanofiber composite scaffold containing silicate-based ceramic particles (Nagelschmidtite, NAGEL, Ca_7_P_2_Si_2_O_16_) through the co-electrospinning technique for diabetic wound healing (Figure 9). In vivo studies revealed that these nanofiber composite scaffolds promoted angiogenesis, the deposition of collagen, and re-epithelialization at the wounded site in the diabetic mice. The results suggested that the release of Si ions and the structure of nanofibrous scaffolds have the potential for diabetic wound healing, and pave the way for biomaterials used in the field of both wound healing and tissue engineering applications [154].

#### 5.2.2. Poly(vinyl alcohol) (PVA)

**Figure 10:**
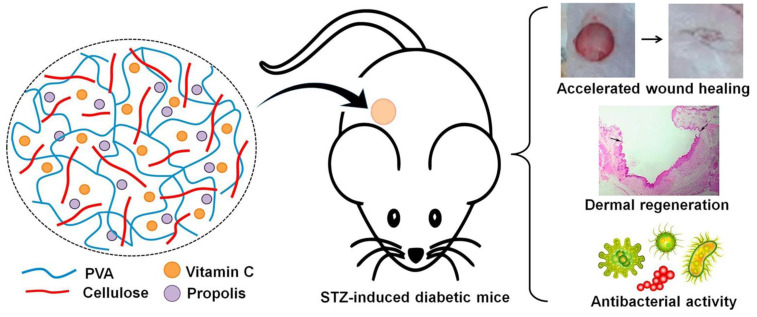
PVA-based films for diabetic wound healing. Reprinted from International Journal of Pharmaceutics, Vol. Number 552 (1–2), Voss G.T., Gularte M.S., Vogt A.G., Giongo J.L., Vaucher R.A., Echenique J.V., Soares M.P., Luchese C., Wilhelm E.A., Fajardo A.R., Polysaccharide-based film loaded with vitamin C and propolis: A promising device to accelerate diabetic wound healing, Pages No 340–351, 2018 with permission from Elseiver [74].

PVA is an excellent biocompatible synthetic polymer produced by the hydrolysis of vinyl acetate. It is one of the US Food and Drug Administration (FDA)-approved synthetic polymeric materials. The value of PVA is growing at an enormous rate, as it is utilized for biomedical applications owing to desirable characteristics such as non-carcinogenic, non-toxic, bio-adhesive, and swelling behaviours. PVA has been potentially used in soft eye lenses, cartilages, and eye drops, etc. PVA can be fabricated in different forms such as fibres, gel, and film that support in aiding the adhesion and proliferation of cells [155]. Huang et al. fabricated electrospun brown alga-derived polysaccharide and PVA nanofibers for skin repair in diabetic mice. Brown alga-derived polysaccharide is a sea mustard found in marine areas. The result suggested that the prepared nanofibers decreased inflammation and stimulated angiogenesis at the wound site of diabetic mice [156]. Lin et al. synthesized PVA/cobalt-substituted hydroxyapatite nanocomposites as a wound dressing material for diabetic foot ulcer treatment. The nanocomposites were prepared using solvent casting method. The result showed that the prepared nanocomposites had high mechanical properties and excellent bioactivity. The nanocomposites discharged a small number of cobalt ions into the cell-cultured medium, which showed better cell growth. The prepared nanocomposites could be a potential wound dressing material for diabetic foot ulcer treatment [157]. Zhu et al. fabricated PVA hydrogel loaded with fibroblast growth factor 21 and metformin for diabetic wound healing. The fabricated hydrogel were injectable, adhesive and ROS scavenging abilities. In vivo results showed the formation of blood vessels with faster healing of diabetic wounds [158]. Wang et al. prepared PVA/chitosan nanocomposite hydrogels incorporated with Tibetan eighteen-flavour dangshen pills (TEP) for treating chronic diabetic wounds. TEP is a traditional Tibetan medicine used to treat skin diseases with analgesic, anti-inflammatory, and healing properties. This hydrogel were treated with L939 cells, which showed no cytotoxic effect and demonstrated that the new formulation can be used for treating diabetic wounds with the help of traditional medicine [159]. Cellulose and PVA-based films incorporated with Vitamin C and/or propolis for faster diabetic wound healing were created by Voss et al. Cellulose-PVA/Vitamin C and Cellulose-PVA/Vitamin C/Propolis films were prepared in order to analyse the release of Vitamin C in a precise manner. When Cellulose-PVA/Vitamin C/Propolis were used in an STZ-induced diabetic animal model, it showed faster wound closure. Histopathological analysis showed better results when treated with Cellulose-PVA/Vitamin-C/Propolis. The results suggest that the prepared PVA-based film could be a potential treatment procedure for faster wound healing (Figure 10) [74].

Ahmed et al. fabricated chitosan, PVA, and zinc oxide nanofibrous mats using electrospinning technique for faster healing of diabetic wounds. The compounds chitosan and PVA have wound-healing properties, and zinc oxide has excellent antibacterial activity. The result showed that these nanofiber mats exhibit efficient antibacterial and antioxidant properties. In vivo analysis showed that there was faster healing of diabetic wounds in groups treated with nanofiber mats [43]. Kim et al. developed a new film-forming hydrogel including PVA, polyvinylpyrrolidone, and propylene glycol incorporated with sodium fusidate for wound healing applications. The film showed excellent elasticity and flexibility, and could be an effective pharmaceutical product for wound treatment [69].

#### 5.2.3. Poly(2-Hydroxyethyl Methacrylate) (pHEMA)

pHEMA is a hydrophilic, non-biodegradable, and biocompatible polymer that is widely used for different types of wound healing, bone regeneration, and cancer treatment. pHEMA-based biomaterial gained lot of attention in wound treatment and ocular therapy due to its excellent biocompatible and minimal thrombogenic properties. Due to its transparent nature, it facilitates the tracking of wound recovery when used as wound dressing material. Bacterial cellulose pHEMA and silver were combined as a multifunctional wound dressing material with efficient antimicrobial properties [160].

#### 5.2.4. Polylactide (PLA) and Polyglycolic Acid (PGA)

PGA and PLA are extensively suitable for the fabrication of scaffolds. These two synthetic polymers serve as a suitable platform for tissue construction. Moreover, these polymers have been used as implantable materials in the field of medicine. Polyglycolic acid is the first synthetic polymer utilized in the form of suture under the name of “Dexon”. At the site of the wound, PLGA and PLA stimulate the supply of lactic acid, and help in inducing angiogenesis and quickening the process of wound healing [155]. Khazaeli et al. prepared PLA/chitosan nanoscaffolds using the microwave-assisted electrospinning technique loaded with cod liver oil for diabetic wound healing. The results showed that the groups treated with nanoscaffolds exhibited wound recovery within 14 days of treatment [161]. Zheng et al. fabricated polylactic co-glycolic acid/cellulose nanocrystal nanofibers loaded with neurotensin to study their therapeutic potency in treating diabetic wounds. The prepared nanofibers were applied on wounds in diabetic mice, which showed a slow release of neurotensin for 2 weeks. The results suggested that the prepared nanofibers effectively stimulate the regeneration of tissues for diabetic foot ulcer treatment [59]. Zha et al. prepared polyglycolic acid/silk fibroin nanofibrous scaffolds incorporated with deferoxamine for diabetic wound healing application. The prepared nanofibrous scaffold had a porous three-dimensional nanofibrous structure and exhibited good mechanical strength, biodegradability, and biocompatibility, which could promote cell adhesion, growth, and migration. The in vivo results showed that the prepared nanofibrous scaffold accelerated the wound healing rate in treated groups [162].

## 6. Future Perspectives and Conclusions

To date, there are numerous polymer-based wound dressings for treating and managing diabetic wounds/foot ulcers. These dressings differ in their porosity, mechanical strength, swelling, and moisture absorption properties. They aid in cellular adhesion, proliferation, and migration without causing any cytotoxicity or immunotoxicity. Wound dressings protect wounds from microbial infections and physical damage. The efficacy of the polymeric biomaterials can be improved by loading therapeutic molecules, growth factors, and anti-microbial agents that could accelerate the process of wound closure by triggering collagen deposition and vascularization. The implantation of the dressing material exactly at the site of the wound is a challenging procedure, as it requires appropriate coordination between the scientist and physician. The method employed to synthesize scaffolds is also crucial as it plays a vital role in determining the quality and performance of the wound dressings. In addition, the fabrication technique may involve costly sophisticated instruments, which may further increase the cost of treatment. Therefore, it is important to recognize the issues that affect clinical translation, and pursue alternatives that can overcome the current problems.

Further, research in the field of 3D printing and tissue engineering can improve the potency of polymeric wound dressings for efficient diabetic wound treatment. Wound dressing materials that are 3D printed can serve as a unique platform and can be incorporated with different types of bioactive compounds and antimicrobial agents that can speed up the rate of wound healing. The use of 3D printing can overcome the disadvantages associated with the conventional techniques, and is highly reliable and low-cost. Additionally, the combination of biomarkers and nanoparticles that can be used to monitor wound recovery can be loaded with the wound dressing materials for efficient diabetic wound treatment.

## Figures and Tables

**Table 1:** Different types of polymeric biomaterial-based dressings.

Authors	Material/Dressings	Therapeutic Compounds	Applications	Ref.
Ahmed et al.	Polyvinyl alcohol—Chitosan nanofiber mats	Zinc oxide NP	Microbial-Infected DW Care	[43]
Cam et al.	Polyvinylpyrrolidone-Polycaprolactone nanofibrous mats	Pioglitazone	DW Healing	[44]
Almasian et al.	Polyurethane—Carboxymethylcellulose nanofibers	Plant extract of *Malva sylvestris*	DW Treatment	[45]
Chen et al.	Poly-N-acetylglucosamine nanofibers	Polydeoxyribonucleotide	Diabetic Skin Ulcer	[46]
Choi et al.	Polyethylene glycol—Polycaprolactone hybrid nanofibers	Human Epidermal Growth Factor	Diabetic Ulcer Treatment	[47]
Cui et al.	Polylactide-based nanofibers	Doxycycline	Chronic Wound Management	[48]
Grip et al.	Hydroxypropyl Methylcellulose/Polyethylene oxide nanofibers	β-Glucan	DW Care	[49]
Kanji et al.	Polyethersulfone nanofibers	Human umbilical cord blood-derived CD34+ cells	DW Management	[50]
Lee et al.	PLGA nanofibers	Platelet-derived growth factor, Vancomycin, and Gentamicin	Diabetic Infected Wound Care	[51]
Lee et al.	PLGA nanofibers	Insulin	DW Recovery	[52]
Merrel et al.	Polycaprolactone nanofibers	Curcumin	DW Management	[53]
Pinzón-García et al.	Polycaprolactone nanofibers	Bixin	DW Healing	[54]
Ranjbar-Mohammadi et al.	Polycaprolactone—Gum Tragacanth nanofibers	Curcumin	DW Care	[55]
Shalaby et al.	Cellulose acetate nanofibers	Silver NP	Microbial-Infected Diabetic Lesion Treatment	[56]
Zehra et al.	Polycaprolactone nanofibers	Sodium Percarbonate	DW Management	[57]
Lee et al.	PLGA—Collagen scaffold membranes	Glucophage	DW Management	[58]
Zheng et al.	PLGA—Cellulose nanocrystals nanofiber membranes	Neurotensin	DW Care	[59]
Liu et al.	Cellulose acetate—Zein composite nanofiber membranes	Sesamol	DW Treatment	[60]
Lee et al.	PLGA membranes	Metformin	DW Healing	[61]
Ren et al.	Poly-L-lactic acid fibrous membranes	Dimethyloxalylglycine-loaded mesoporous silica NP	DW Treatment	[62]
Lobmann et al.	Hyaluronic acid membranes	Human keratinocytes	Diabetic Foot Wounds	[63]
Augustine et al.	Poly(3- hydroxybutyrate-co-3-hydroxyvalerate) membranes	Cerium oxide NP/gelatin	DW Treatment	[64]
Augustine et al.	Polyvinyl alcohol—Polylactic acid hybrid membranes	Connective tissue growth factor	Wound Dressing Membranes For Diabetic Lesions And Chronic Ulcers	[65]
Arantes et al.	Chitosan films	Retinoic acid / solid lipid nanoparticles	DW Healing	[66]
Arul et al.	Collagen films	Biotinylated GHK peptide	DW Dressing	[67]
Inpanya et al.	Fibroin films	Aloe gel	DW Management	[68]
Kim et al.	Polyvinylpyrrolidone—Polyvinyl alcohol films	Sodium fusidate	Wound Healing	[69]
Mizuno et al.	Chitosan films	Fibroblast growth factors	DW Healing	[70]
Song et al.	Cellulose films	Selenium	Cutaneous DW Healing	[71]
Tan et al.	Sodium alginate hydrocolloid films	Vicenin-2	DW Management	[72]
Tong et al.	Polyvinyl alcohol—Cellulose anocrystal films	Curcumin	DW Care	[73]
Voss et al.	Cellulose—Polyvinyl alcohol films	Propolis and/or Vitamin C	DW Management	[74]
Wu et al.	Silk Fibroin—Chitosan films	Adipose-derived stem cells	DW Care	[75]
Da Silva et al.	Hyaluronic acid spongy hydrogels	Human adipose stem cells	Diabetic Foot Ulcer	[76]
Lai et al.	Sodium carboxymethylcellulose hydrogels	Fern extracts *(Blechnum orientale Linn.)*	Diabetic Ulcer Treatment	[77]
Li et al.	Hydroxyapatite/Chitosan composite hydrogels	Exosomes (SMSCs-126)	DW Treatment	[78]
Masood et al.	Chitosan—Polyethylene glycol hybrid hydrogels	Silver NP	DW Healing	[79]
Shi et al.	Chitosan—Dextran hydrogels	Silver NP	DW Treatment	[80]
Thangavel et al.	Chitosan hydrogels	L-glutamic acid	DW Healing	[81]
Zhang et al.	Poly (γ-glutamic acid)—Heparin—Chitosan composite hydrogels	Superoxide dismutase	DW Treatment	[82]
Choi et al.	Polyurethane foams	Silver nanoparticles and Recombinant Human Epidermal Growth Factor	Bacteria-Infected DW Management	[83]
Pyun et al.	Polyurethane foams	Recombinant Human Epidermal Growth Factor	DW Treatment	[84]
Atia et al.	Sodium alginate—Gelatin wafers	Diosmin nanocrystals	DW Healing	[85]
Anisha et al.	Hyaluronic acid—Chitosan sponges	Silver nanoparticles	Wound Dressing for Diabetic Foot Ulcer	[86]
Lipsky et al.	Collagen sponges	Gentamicin	Diabetic Foot Ulcer	[87]
Mohandas et al.	Chitosan—Hyaluronic acid composite sponges	Fibrin nanoparticles incorporated with vascular endothelial growth factors	Wound Dressing For DW	[88]
Shi et al.	Chitosan—Silk hybrid sponges	Gingival mesenchymal stem cell-derived exosomes	DW Healing	[89]
Wang et al.	Chitosan—Collagen sponges	Recombinant Human Acidic Fibroblast Growth Factors	DW Healing	[90]
Xia et al.	Chitosan composite sponges	Quaternary ammonium chitosan nanoparticles	Wound DressingMaterial for Diabetic Chronic Injury	[91]
Kondo et al.	Hyaluronic acid—Collagen sponges	Epidermal growth factors	DW Healing	[92]
Raveendran et al.	Chitosan bandages	Ciprofloxacin and Fluconazole-containing Fibrin nanoparticles	DW Management	[93]
Mohanty et al.	Sodium alginate—Chitosan bandages	Epidermal growth factor, curcumin, and mesenchymal stem cells	DW Healing	[94]
Kumar et al.	Chitosan hydrogel composite bandages	Zinc oxide nanoparticles	Wound Dressing Material	[95]

NP—nanoparticles; PLGA—polylactic-co-glycolic acid; DW—diabetic wound.

## Data Availability

Not applicable.
